# Changes in clinicomorphometrical findings, lipid profiles, hepatorenal indices and oxidant/antioxidant status as thermoregulatory adaptive mechanisms in poikilothermic Dabb lizard (*Uromastyx aegyptia*)

**DOI:** 10.1038/s41598-023-30184-z

**Published:** 2023-02-28

**Authors:** Eman A. R. Abdelghffar, Ameera G. ALmohammadi, Samina Malik, Arafat Khalphallah, Mohamed Mostafa Soliman

**Affiliations:** 1 Department of Zoology, Faculty of Science, Ain Shams University, Abbasseya, Cairo, 11566 Egypt; 2 Department of Biology, College of Science, Taibah University, Yanbu, Kingdom of Saudi Arabia; 3 University College of Medicine and Dentistry, University of Lahore, Lahore, Pakistan; 4 Division of Internal Medicine, Department of Animal Medicine, Faculty of Veterinary Medicine, Assiut University, Assiut, 71526 Egypt; 5 Biology Department, Faculty of Science, Jazan University, Jazan, Kingdom of Saudi Arabia

**Keywords:** Zoology, Herpetology

## Abstract

Wildlife has exposed to various environmental stressors. Reptiles (ectothermic) are highly susceptible to climatic changes due to their behaviour, physiology, and life history that were so heavily reliant on the ambient environmental temperature. The present work aims to monitor different biochemical and haematological indices of Dabb lizards (*Uromastyx aegyptia*) at various thermal gradients as well as their adaptation to oxidative stress. This has been reflected through assessment of their impact on some adaptive physiological traits i.e. thermoregulation, and muscle metabolic biomarkers, blood pictures and oxidant/antioxidant status. This experiment is carried out on non-hibernating adult male Dabb lizards (*U. aegyptia*; n = 24) of age of 18–24 months. These Dabb lizards are divided into four equal groups (n = 6 for each one) where they are exposed to different thermal treatments for one week as following; control group [Exposed to terrarium temperature 38–39 °C], low temperature exposed group [Exposed to 12–14 °C], Gp. C; moderate temperature exposed group [Exposed to 41–43 °C] and high temperature exposed group [Exposed to 43–45 °C]. Each independent group (n = 6) are kept at separated glass terraria. The investigated lizards are monitored for body temperature, morphometric measurements i.e. body weight (g) and total body length (cm; TBL), muscle biochemical analysis, haematological pictures indices and serum biochemical assays including mainly oxidant/antioxidants biomarkers throughout the current experiment. The results state that the thermoregulatory behaviour of Dabb varies with the increase of concentration of muscular metabolic enzymes. In low temperature exposed group, the increase in red blood corpuscles (RBCs), haemoglobin concentrations (Hb), white blood cell (WBC), serum antioxidant biomarkers and anaerobic Lactate dehydrogenase (LDH) enzyme are associated with a marked reduction in serum levels of total cholesterol (TC), triglycerides (TGs), total proteins (TPs), albumin, glucose and electrolytes. In moderate temperature exposed group, a significant elevation in serum values of TC, TGs, TPs, glucose, urea and uric acids levels are mentioned. In high temperature exposed Dabb group, a remarkable increase in blood values of RBCs, Hb, haematocrit value (HCT), WBC, T. chol., TGs, TPs, glucose, urea, uric acids, triiodothyronine (T3) and thyroxine (T4) levels are also observed. Moreover, significant increases in muscular anaerobic/aerobic metabolic enzymes as well as stimulation of antioxidant defence system have been reported. Different significant correlations have been stated between variably estimated laboratory indices in the investigated Dabb lizards under different thermal treatments. The study concludes that the Dabb lizards have a strong antioxidant defence system and undergo physiological thermoregulatory adaptive mechanisms, that involve biochemical and metabolic acclimatization as a response to environmental temperature changes that act as a protective mechanism against oxidative stress as well as maintained homeostatic responses and normal physiological functions.

## Introduction

Wildlife is usually exposed to many environmental stressors such as extreme/sudden weather abnormalities in addition to human activities. Ectotherms may be particularly affected by these climatic changes because of their restricted ability to utilise metabolic heat to regulate body temperature^1^.

Worldwide species have faced unlimited challenges because of the current global climate change. It is greatly important for scientists and conservation managers to comprehend how the climate change will affect natural populations^2^. Considering this, the ectotherms have thermal adaptation to the climatic changes because of their extraordinary sensitivity of their physiology and life history to temperature change^2^ as well as changing their behaviour by changing either their thermal needs or thermal tolerances^3^.

Reptiles are ectothermic animals, meaning that a large number of their physiological processes (e.g. growth, reproduction, speed of movement, digestion, and metabolism) are significantly affected by thermal changes in the environment and this refers clearly to their great dependence on ambient temperature. Reptiles in moderately seasonal climates can retain activity through a combination of metabolic ability compensation and/or thermoregulation behaviour at molecular and cellular levels after the winter months^4–8^ to help them deal with stressors and maintain homeostasis.

The *Uromastyx aegyptia aegyptia* (FORSKAL 1775; *U. a. aegyptia*) lizard is one of the most common species in North Africa like Egypt and the Middle East like Saudi Arabia and other countries^9,10^. The behavioural and physiological characteristic of *U. aegyptia* like the other ectotherms is influenced by body temperature. Reptiles, for instance, can frequently create both short- and long-term acclimation reactions in response to changes in the environmental temperature to better match their physiological performance to the regional thermal circumstances^2,3^.

*Uromastyx aegyptia* (*U. aegyptia*), like as others *Uromastyx species*, is primarily herbivorous but occasionally eats beetles, ants, grasshoppers, and even scorpions, especially when young (as an omnivorous), depending on seasonal variation in food resources^11^.

*U. aegyptia* preferred body temperature may be very similar to that of a closely related species, *Uromastix acanthinurus*, which prefers a body temperature of 40 °C^12^. In spring and summer period, *U. acanthinurus* internal temperatures can reach the maximum voluntarily tolerated, close to 45 °C^12^, which is the highest temperature that can be deliberately sustained. In April they vary between 33 and 39 °C and in June they are most often between 35 and 42 °C, During winter, its metabolism remains very low at temperatures below 35° C (reach to 10–14 °C in their burrows), would mean that, physiologically, they would have been under their thermal optimum and in a state of metabolic depression^13^.

Models’ future projections reveal that some parts of Africa i.e. mainly west and center, and the Arabian Desert will become climatically unsuitable for *Uromastyx* species (e.g., *U. dispar*, *U. geyri*, *U. acanthinura*, *U. nigriventris*, *U. thomasi*). They also suggest that *Uromasty* species will extend their latitudinal ranges between periods, mainly through northward shifts of their current ranges^14^.

Temperature oftenly has a significant impact on mitochondrial functions. In warmer habitats, greater mitochondrial activity induces faster development in ectotherms. This may lead to smaller body size and higher rates of oxidative stress^1^. Rapid development has already required the more production of adenosine triphosphate (ATP) in the mitochondria and this can induce reactive oxygen species (ROS) generation. ROS have many advantageous physiological functions, such as maintaining homeostasis and cell signaling^15,16^. But when ROS level sometimes exceeds the ability of cells to detoxify them with antioxidants, ROS usually cause oxidative damage to vital biomolecules like membrane lipids, proteins, and DNA, and ultimately the entire organism ages more quickly as a consequence for this damage^16,17^. Also, mitochondrial function is likely to be restricted during thermal extremes, especially in ectothermic animals^8,17^.

During brumation or hibernation or aestivation, decreased aerobic capacity and decreased oxygen consumption cause the partial pressure of oxygen to rise to a critical level, where electron accumulation at the mitochondrial electron transport chain results in the generation of ROS, which results in oxidative stress^1,16^. For example, Ali et al.^18^ note metabolic decline and enhanced ROS generation by isolated mitochondria at moderately lower temperatures.

Mitochondria is the key part in metabolism and skeletal muscle function. Mitochondrial function impairments are regularly connected with degenerative infections. Citrate synthase enzyme (CS) is a key enzyme of the matrix mitochondrial in the glucose oxidation (citric acid cycle; CAC) and its activity is regularly used as a biomarker of mitochondrial substance and capacity^5,8^.

Reptilian thyroid hormones usually fluctuate within a yearly cycle in association with activities like brumation and reproduction as a result of oscillations in climate. Indeed, researchers reveal that the axis that has been constituted by the hypothalamus, pituitary gland, and thyroid, has been varied in reptiles according to season^19,20^. Sciarrillo et al.^20^ report a very pronounced annual cycle of blood thyronine concentration in the male lizard (*Podarcis sicula*), which reaches maximum levels during the hottest periods of the year then decline significantly in winter.

Moreover, reptiles have extremely diverse thyroid function responses after being exposed to cold, reflective of their various thermoregulatory strategies. They display a decrease in thyroid function as well as metabolism in response to the cold exposure^21,22^. But when exposed to warmer temperatures, poikilotherms' thyroid activity increases^21–23^.

Changes in haematological measurements may reflect environmental change, and presence of diseases, as well as they are crucial in assessing the health of organs^24^. The ability of red blood corpuscles (RBCs) to counteract oxidative stress is critically dependent on glucose-6-phosphate dehydrogenase (G6PDH). G6PDH is the only source of nicotinamide adenine dinucleotide phosphate (NADPH as reducing energy) in the pentose phosphate pathway, which in turn is critical in preserving high cellular levels of reduced glutathione (GSH) to save the cell from damage induced by oxidative stress and to maintain redox balance. Therefore, it has a major necessary role in defending against oxidative damage^25^.

No much available information is reported about the physiology of the Dabb lizard (*U. aegyptia*) especially about antioxidants system and mitochondrial enzymes related to energy metabolism. Accordingly, the present work aims to monitor different biochemical i.e. serum and muscular, and haematological assays of Dabb lizards (*Uromastyx aegyptia*) at various thermal grades as well as their adaptation to oxidative stress that are reflected through assessment of some adaptive physiological traits (Thermoregulation), blood pictures, blood lipid profile, hepatorenal function, thyroid functions, serum oxidant/antioxidant status and muscle metabolic biomarkers.

## Materials and methods

### Ethics approval and consent to participate

The authors confirmed that study was reported in accordance with ARRIVE guidelines (https://arriveguidelines.org) whereas the experimental protocol was approved by ethical committee of Faculty of Science, Ain Shams University licensed number ASU-SCI/ZOOL/2022/11/4 and that of Faculty of Veterinary Medicine, Assiut University, Egypt licensed number 06/2023/0038 which are in accordance with the international reference standards for the guide to the use of animals in various research experiments and their care without prejudice to laws and ethics which organizes the conduct of various scientific research experiments.

### Animals and experimental design

**Figure 1:**
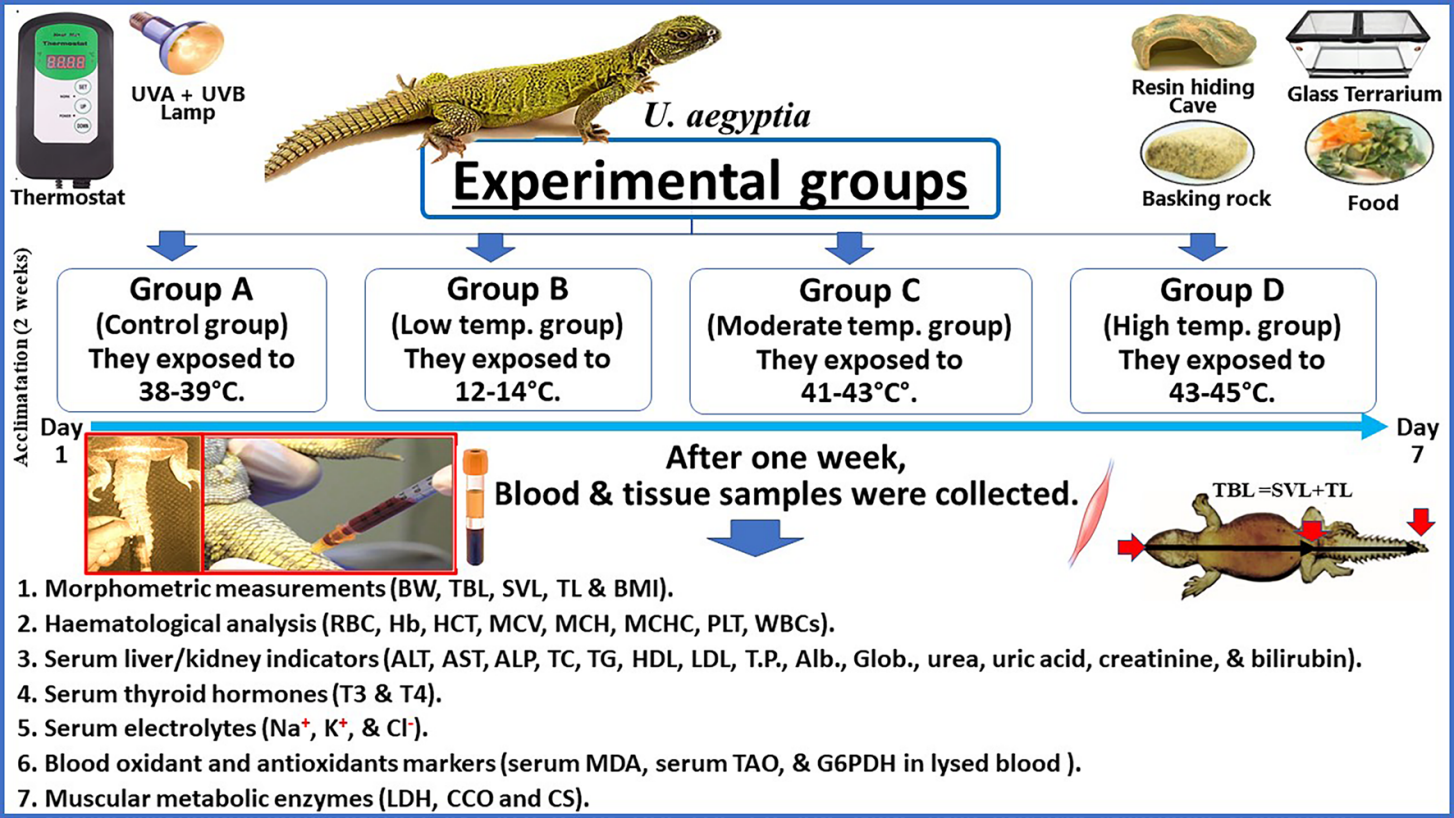
It represented experimental design and measuring parameters.

The experiments were carried out between April and May 2018 (Spring season). This experiment was carried out on non-hibernating adult male Dabb lizards (*U. aegyptia*; n = 24) of age of 18–24 months. The main site of captive Dabb lizards from Talaba Abdel Halim farm, Abu Rawash village, Kerdasa Center, Giza Governorates, Egypt. The Dabb lizards were individually housed in glass terraria with sandy substrate, large rock for basking, resin rock Cave for hiding, overhead lamps (MIXJOY UV-A/UV-B Sun Lamp, 160 watts) that gave both heat/light that turned off at night and were set for a light/dark cycle of 12 h and fed on corn seeds and leaf lettuces, as well as bits of zucchini and carrots. Water was provided ad libitum. They were given two weeks for acclimation prior to start the experiment (Fig. 1).

These Dabb lizards were divided into four equal groups; control group, low temperature exposed group, moderate temperature exposed group and high temperature exposed group. Each group (n = 6) of them (n = 24) were kept at glass terraria. Control group was exposed to Terrarium temperature (38–39 °C) and kept as control group. The animals in low, moderate and high temperature exposed Dabb lizards’ groups were exposed to low (12–14 °C), moderate (41–43 °C), and high (43–45 °C) temperatures, respectively, for one week. Low and high temperatures were chosen based on seasonal changes in the winter, and summer seasons in their natural range.

At low temperatures (12–14 °C), blood sampling was performed after the lizards were kept for a week on a laboratory refrigerator (PHCbi, formerly Panasonic, MPR-722R-PA, Series 23.7 Cu. Ft., its temperature range: 2–23 °C) with temperature of 12–14 °C. At moderate temperature, the glass terraria containing the lizards were introduced in a water bath (Gallenkemp BKS-350-010Q) at 41–43 °C. At high temperature, the glass terraria with the lizards were again introduced in a water bath but at a temperature of 43–45 °C.

In the laboratory, the body temperature of the lizards was monitored using ultra-thin thermocouple probes that were linked to tele-thermometer (YSI Tele-thermometer, Model 44-Twelve channel, Yellow Springs Instrument Co., Inc., Ohio) alongside an Omni scribe Houston Instrument B5217-1 chart recorded for keeping track of inner body temperature. The thermocouple probes (Temperature sensors) were embedded within the cloaca of lizards during the run. The length of the wire attached was long enough to allow the lizards to move freely in the laboratory at a given temperature stated above.

### Morphometric measurements

Body weight (g) before and after the experiments as well as total body length (cm; TBL) of captured male Dabb lizards were measured. The TBL was calculated by measuring the snout-vent length plus the tail length using a measuring tape. A body-mass-index (BMI) was computed for each animal using the following equation: [BMI = final body weight (g)/TBL (cm)] according to following^11^.

### Sampling

At the end of experiment, the low and high temperature treatment did not affect the survival rate of the Dabb lizards. The lizards were anaesthetised with Ketamine (10 mg/kg, IM; recovery 3–4 h)^26^ to restrain the lizard. About 2 ml of blood samples were collected from the ventral coccygeal vein with or without ethylene diamine tetra-acetic acid (EDTA). 0.5 ml blood plus EDTA (1.5 mg/ml blood) was used to measure haematological parameters. Another portion of blood (1.5 ml) without anticoagulant was used to estimate the biochemical indices. Then the blood samples were centrifuged (4000 rpm for 20 min) and the serum samples (≈ 0.5 ml) were collected into Eppendorf tubes, then kept frozen for one day at − 20 °C for subsequent serum biochemical analyses including lipid profiles, hepatorenal functions, thyroid functions and serum oxidant/antioxidant status using commercial test kits according to the standard protocols of suppliers^27^. In addition, the tail muscle tissue samples (tail tip: a non-lethal tissue sample) were collected and homogenised in 50 mM cold sodium phosphate buffer (pH 7.0) at a ratio of 1:10 (w/v), followed by centrifugation at 15,000 rpm for 20 min at 4 °C. After collection, the supernatant was stored at − 80 °C to await muscle biochemical analysis.

### Complete blood picture indices

Complete blood picture including RBCs, white blood cells (WBC), haemoglobin concentration (Hb) and haematocrit value (HCT) and platelets (PLT), were assayed by Coulter counter (Nihon Koden Celltac *alpha* MEK-6400 series, Tokyo, Japan). Mean corpuscular values such as mean corpuscular volume (MCV), mean corpuscular haemoglobin (MCH), and mean corpuscular haemoglobin concentration (MCHC), were estimated according to Harvey^28^; Latimer et al.^29^.

### Serum Lipid profile indices

The Spectro Ultraviolet-Vis RS spectrophotometer (Labomed, Inc., Los Angeles, CA, USA) was used to determine serum concentrations of serum levels of glucose (CAT.NO. GL364), total cholesterol (TC; CAT.NO. CH201), triglycerides (TGs; CAT.NO. TR213), high-density lipoproteins (HDL; CAT.NO. CH1383), low-density lipoproteins (LDL; CAT.NO. CH2656), total proteins (TPs; CAT.NO. TP245) and albumin (CAT.NO. AB362), were assessed by using commercial diagnostic kits (Randox Laboratories, UK) according to manufacturer’s instructions. Serum globulins were estimated by subtraction of albumin from TPs and their values used to calculate albumin/globulin ratio (A/G ratio). Atherosclerosis indices were also calculated.

### Serum hepatorenal biomarkers and electrolytes indices

Hepatorenal indicators were determined spectrophotometrically using Spectro Ultraviolet-Vis RS spectrophotometer (Labomed, Inc., Los Angeles, CA, USA) through the commercially supplied diagnostic kits (Randox Laboratories, UK) according to manufacturer’s instructions. These biomarkers included alanine transaminase (ALT; CAT.NO. AL1205), aspartate aminotransferase (AST; AS3804), alkaline phosphatase (ALP; CAT.NO. AP9764) and bilirubin (CAT.NO. BR2361) for hepatic function assessment while they included urea (CAT.NO. UR1068), uric acid (CAT.NO. UA230), creatinine (Cr; CAT.NO. CR2336), potassium (K^+^) (CAT.NO. PT3852), and sodium (Na^+^) (CAT.NO. NA3851) for renal function and electrolytes assessment. Serum chloride (CAT.NO. C501-480) was measured by Teco laboratory kit (Teco Diagnostics, CA, USA).

### Serum thyroid functions indicators

Thyroid functions indictors including free thyroxine (FT4; CAT.NO. OCFD03-FT4-K) and free triiodothyronine (FT3; CAT.NO. OCFH57- FT3) were determined using radioimmunoassay (RIA) kits (Cisbio Bioassays, Codolet, France).

### Serum Oxidant and antioxidant analysis

Serum malondialdehyde (MDA; CAT.NO. E-BC-K025-S) and total antioxidant status (TAO; CAT.NO. NX2332) levels were measured spectrophotometrically using Spectro Ultraviolet-Vis RS spectrophotometer (Labomed, Inc., Los Angeles, CA, USA) with commercially available kits (Elabscience, US) and (Randox Laboratories, UK), respectively. Glucose-6-phosphate dehydrogenase (G6PDH; CAT.NO. PD410) was measured with commercial diagnostic kits (Randox Laboratories, UK) through using quantitative spectrophotometric assay.

### Muscle biochemical analysis i.e. muscle metabolic biomarkers

In supernatant of skeletal muscle, lactate dehydrogenase (LDH; CAT.NO. L3916), and citrate synthase (CS; CAT.NO. CS0720) levels of skeletal muscle were measured using commercially available kits (Sigma-Aldrich, USA), as well as cytochrome c oxidase (CCO; CAT.NO. CB008) was determined using commercially kit (Cell Biologics Company, Chicago, USA) according to manufacturer’s instructions.

### Statistical analysis

Computer Software (SPSS version 16.0, Chicago, USA) was used to analyse all obtained data. Data were shown as mean ± standard error (SE) values. The data obtained from morphometric and laboratory analyses were analysed by general linear model repeated measures ANOVA, and the significance level of results was set at *p* < 0.05. The significance of differences was evaluated between the means at control, low, moderate and high temperature exposed Dabb lizards’ groups. Correlation coefficient was calculated using Pearson Correlation at *p* < 0.05 or *p* < 0.01 between different obtained laboratory results in different Dabb lizards’ groups.

### Institutional review board statement

All animal procedures performed in this study were done according to ethical committee of Faculty of Science, Ain Shams University licensed number ASU-SCI/ZOOL/2022/11/4 and that of Faculty of Veterinary Medicine, Assiut University, Egypt licensed number 06/2023/0038 which are in accordance with the international reference standards for the guide to the use of animals in various research experiments and their care without prejudice to laws and ethics which organizes the conduct of various scientific research experiments.

## Results

### Clinical findings and morphometric measurements

Clinical findings and morphometric measurements including body weight, TBL (SVL plus TL) and BMI were not significantly changed in all investigated groups compared to control group (Table 1).

**Table 1:** The morphometric measurements in the different experimental groups of Dabb lizard (*U. aegyptia*).

Parameters	Experimental groups			
	Group A	Group B	Group C	Group D
Initial BW (g)	1088 ± 60.3	1057 ± 60.6	1122 ± 64.4	1080 ± 66.7
Final BW (g)	1093 ± 59.6	1061 ± 59.6	1128 ± 63.4	1085 ± 65.95
BW gain (g)	5.6 ± 1.0	4.4 ± 1.1	5.5 ± 1.1	5.2 ± 1.0
SVL (cm)	22.6 ± 0.8	23.7 ± 0.7	23.7 ± 0.3	23.2 ± 0.4
TL (cm)	17.8 ± 0.8	18.3 ± 0.4	17.8 ± 0.7	18.8 ± 0.7
TBL (cm)	40.4 ± 1.6	42.0 ± 1.1	41.5 ± 1.0	42.1 ± 1.1
BMI	27.2 ± 1.5	25.3 ± 1.5	27.1 ± 2.0	25.8 ± 1.4

Data are expressed as mean ± SE.

*SVL* Snout-vent, *TL* Tail length, *TBL* Total body length, *BMI* Body-mass-index, *Group A* Control group, *Group B* Low temperature exposed group, *Group C* Moderate temperature exposed group, *Group D* High temperature exposed group, *BW* Body weight.

### Complete blood picture indices

**Figure 2:**
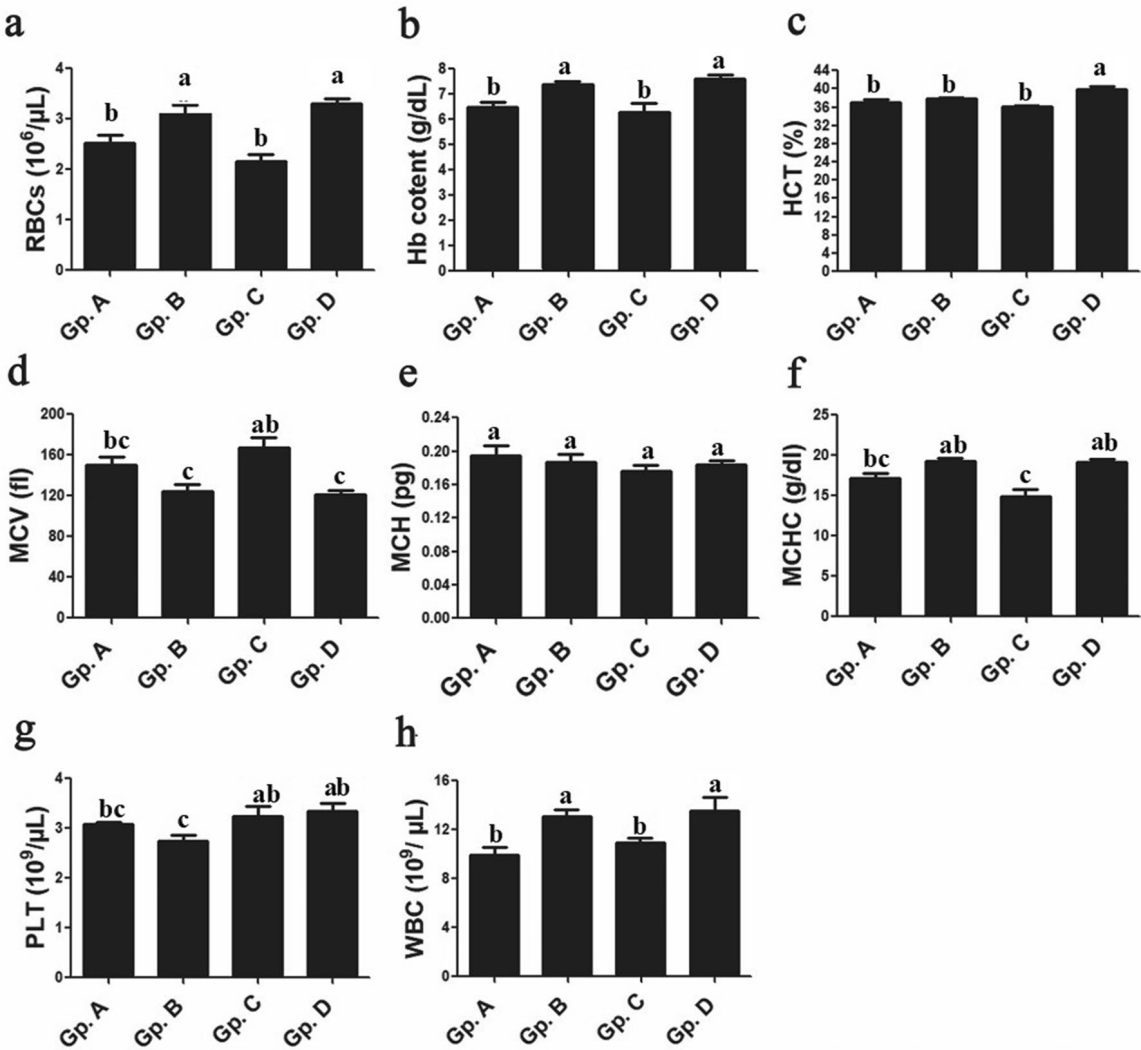
Effect of temperature changes on some haematological parameters (**a**: RBCs; **b**: Hb; **c**: HCT; **d**: MCV; **e**: MCH; **f**: MCHC; **g**: PLT; **h**: WBC) in the different experimental groups of Dabb lizard (*U. aegyptia*). Data were expressed as mean ± SE. Gp. A: control group; Gp. B: low temperature exposed group; Gp. C: moderate temperature exposed group; Gp. D: high temperature exposed group; RBC: red blood corpuscles; Hb: haemoglobin concentrations; HCT: haematocrit value; MCV: mean corpuscular volume; MCH: mean corpuscular haemoglobin; MCHC: mean corpuscular haemoglobin concentration; PLT: platelets; WBC: white blood cell. ^abc^Means above each bar with different letters in different Dabb groups i.e. Gp. A, Gp. B, Gp. C and Gp. D, were significantly different (P < 0.05).

All blood pictures parameters in moderate temperature exposed group were not remarkably changed comparing to control group. All investigated Dabb lizards group had no significant changes between control group and other thermal Dabb treated groups, for PLT and mean corpuscular values i.e. MCV, MCH and MCHC. Mean values of RBCs, Hb and WBC were remarkably (*P* < 0.05–0.001) raised in low temperature exposed group and high temperature exposed group comparing with those in control group. These significant changes (*P* < 0.05) were reported for HCT only between high temperature exposed group and control group while they were absent between control Dabb lizards group and low temperature exposed group (Fig. 2).

### Serum Lipid profile indices

**Figure 3:**
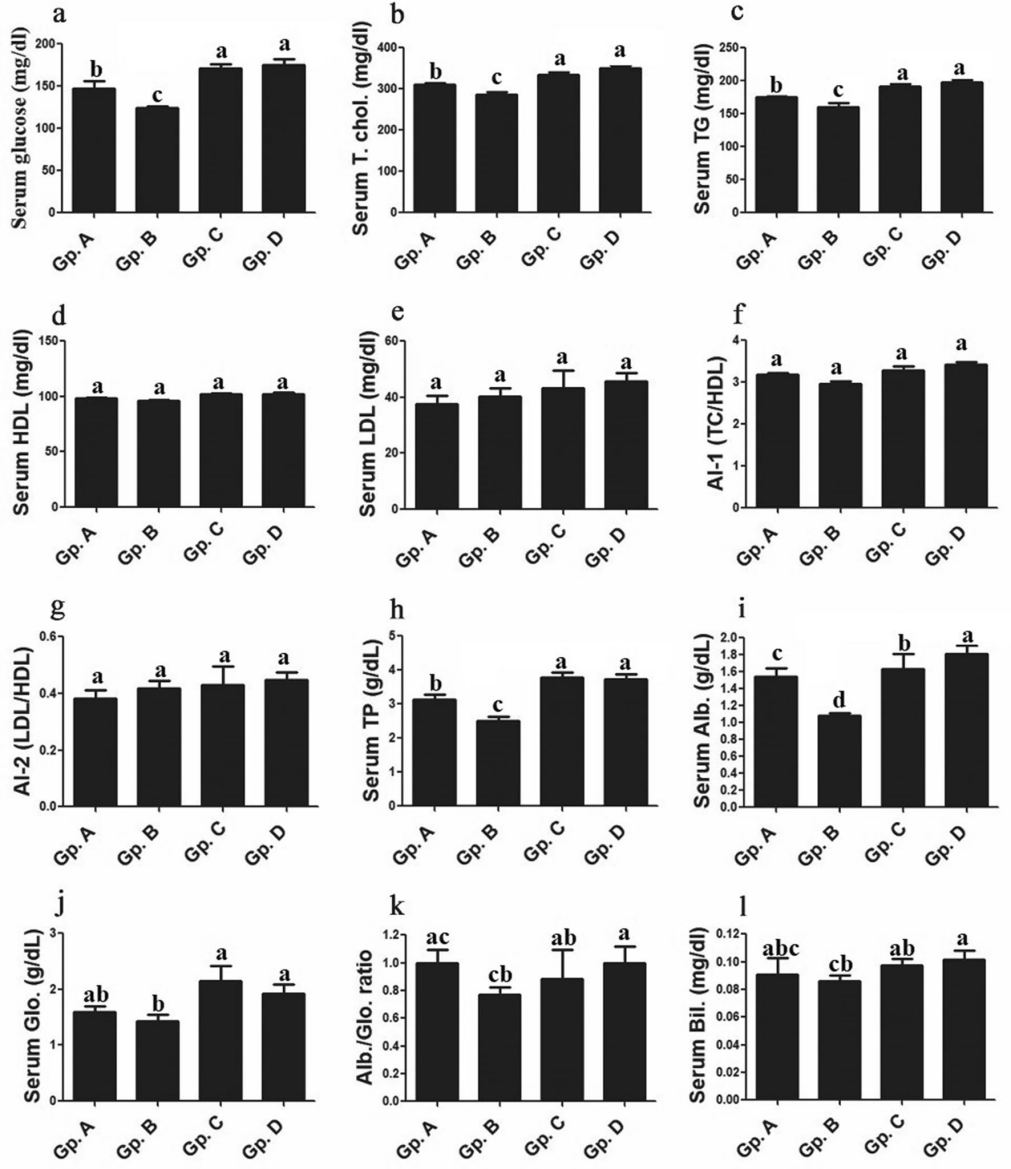
Effect of temperature changes on some biochemical parameters (**a**: glucose, **b**: T. chol.; **c**: TG; **d**: HDL; **e**: LDL; **f**: AI-1; **g**: AI-2; **h**: TP; **i**: Alb.; **j**: Glo.; **k**: Alb./Glo. Ratio, & **l**: Bil.) in the different experimental groups of Dabb lizard (*U. aegyptia*). Data were expressed as mean ± SE. Gp. A: control group; Gp. B: low temperature exposed group; Gp. C: moderate temperature exposed group; Gp. D: high temperature exposed group; TC: total cholesterol; TG: triglyceride; HDL: high-density lipoprotein; LHD: low-density lipoprotein; AI-1: atherogenic index 1 (TC/HDL); AI-2: atherogenic index 2 (LDL/HDL); TP: total protein; Alb.: albumin; Glo.: globulin; Bil.: bilirubin. ^abcd^Means above each bar with different letters in different Dabb groups i.e. Gp. A, Gp. B, Gp. C and Gp. D, were significantly different (*P* < 0.05).

Serum Lipid profile indices showed no significant changes between different Dabb lizards groups either for HDL, LDL, atherogenic index (TC/HDL and LDL/HDL), globulin or for A/G ratio. Different thermal treated Dabb groups i.e. low, moderate and high temperature exposed groups, showed remarkably changes for blood glucose, TC, TGs, TPs and albumins comparing to control group. Significant (*P* < 0.05) reductions of serum glucose, TC, TGs, TPs and albumins were reported in low temperature exposed group when compared with control group. In contrast, moderate and high temperature exposed Dabb groups had significantly (*P* < 0.05–0.001) higher values of blood glucose, TC, TGs, TPs and albumins than those in control Dabb group (Fig. 3).

### Serum hepatorenal biomarkers and electrolytes indices

**Figure 4:**
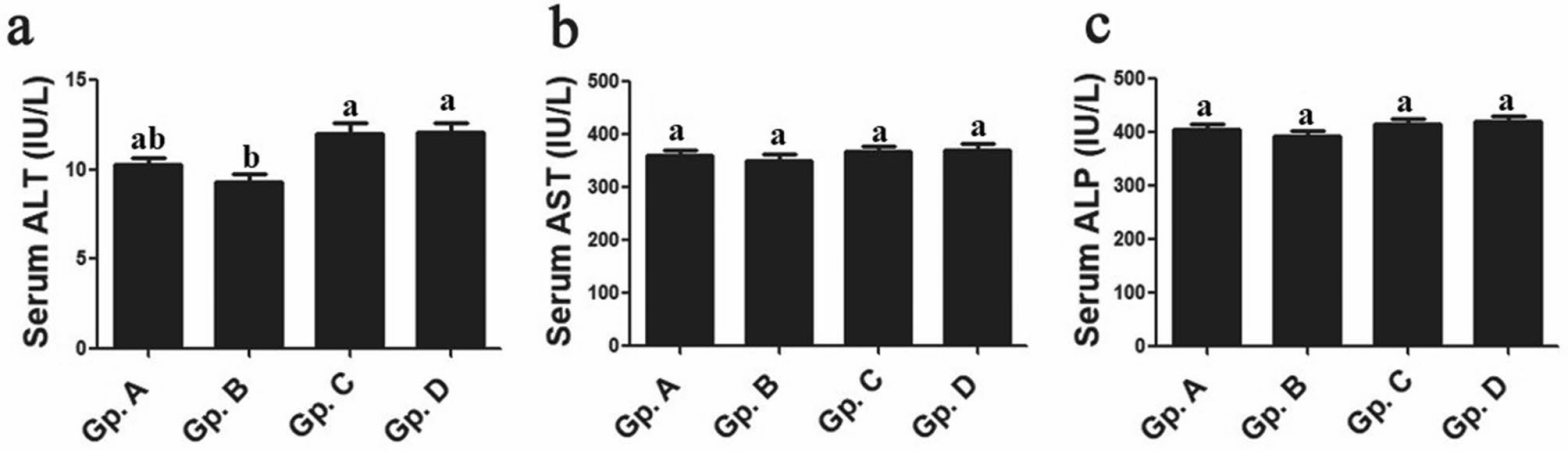
Effect of temperature changes on serum hepatic enzymes markers (**a**: ALT; **b**: AST; **c**: ALP) in the different experimental groups of Dabb lizard (*U. aegyptia*). Gp. A: control group; Gp. B: low temperature exposed group; Gp. C: moderate temperature exposed group; Gp. D: high temperature exposed group; ALT: alanine aminotransferase; AST: aspartate aminotransferase; ALP: alkaline phosphatase. ^ab^Means above each bar with different letters in different Dabb groups i.e. Gp. A, Gp. B, Gp. C and Gp. D, were significantly different (*P* < 0.05).

The current work did not report significant variations for hepatic functions indicators whereas serum activities and values of bilirubin, ALT, AST and ALP were not remarkably altered between control group and other thermal treated Dabb lizards’ groups either low, moderate or high temperature exposed group (Fig. 4).

**Figure 5:**
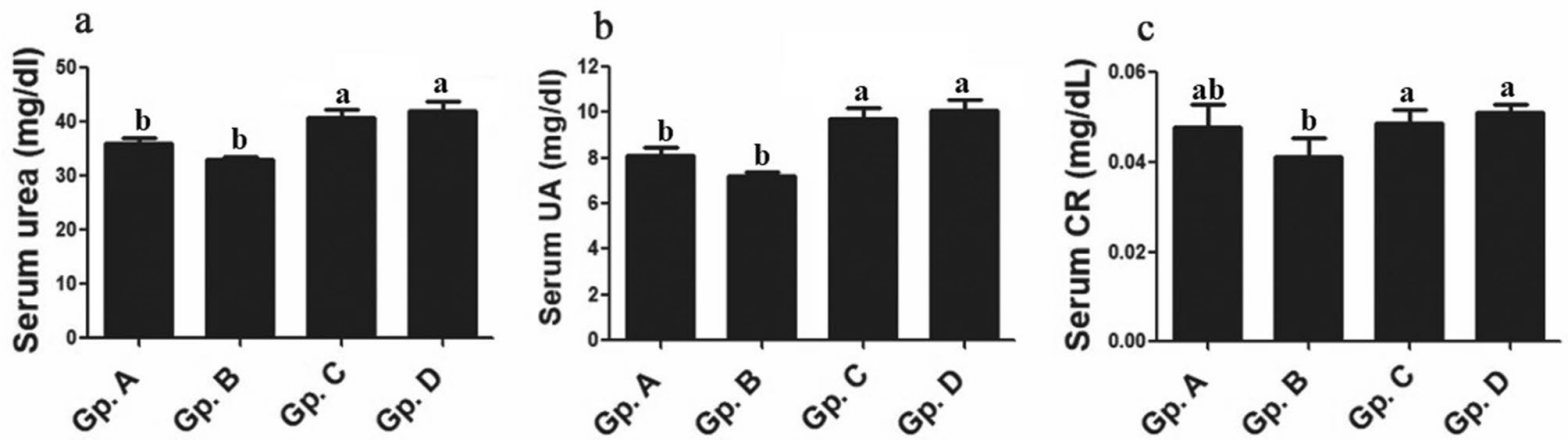
Effect of temperature changes on serum kidney function (**a**: urea; **b**: UA; **c**: CR) in the different experimental groups of Dabb lizard (*U. aegyptia*). Gp. A: control group; Gp. B: low temperature exposed group; Gp. C: moderate temperature exposed group; Gp. D: high temperature exposed group; CR: creatinine; UA: uric acid. ^ab^Means above each bar with different letters in different Dabb groups i.e. Gp. A, Gp. B, Gp. C and Gp. D, were significantly different (*P* < 0.05).

The renal biomarkers showed significant alterations for serum urea and uric acid between control group, and each of moderate and high temperature exposed groups meanwhile these changes were absent between control group and low temperature exposed group. Serum concentrations of urea and uric acid were significantly (*P* < 0.05) elevated in moderate and high temperature exposed groups comparing with their values in control Dabb group. On other hand, serum concentrations of Cr had no significant variations between control groups and other thermal treated Dabb groups (Fig. 5).

**Figure 6:**
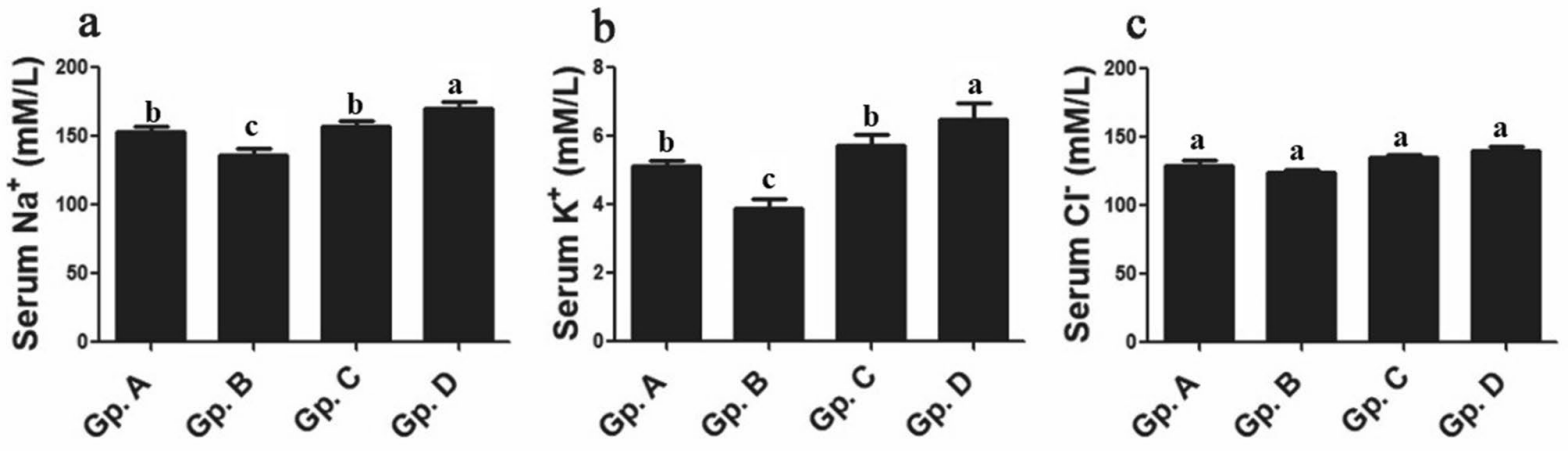
Effect of temperature changes on electrolyte’s parameters (**a**: Na^+^; **b**: K^+^ & **c**: Cl^−^) in the different experimental groups of Dabb lizard (*U. aegyptia*). Gp. A: control group; Gp. B: low temperature exposed group; Gp. C: moderate temperature exposed group; Gp. D: high temperature exposed group; Na^+^: sodium; K^+^: potassium; Cl^−^: chloride. ^abc^Means above each bar with different letters in different Dabb groups i.e. Gp. A, Gp. B, Gp. C and Gp. D, were significantly different (*P* < 0.05).

Serum electrolytes values had significant changes for Na^+^ and K^+^ between control group, and each of low and high temperature exposed groups, however, these significant variations were not observed between control group and moderate temperature exposed Dabb group. Significant elevations (*P* < 0.05) in blood concentrations of Na^+^ and K^+^ in high temperature exposed Dabb group while they were significantly (*P* < 0.05) dropped in low temperature exposed group comparing with their control group values. Moreover, no remarkable changes were stated between control Dabb lizards and the other treated groups for serum values of chloride (Fig. 6).

### Serum thyroid functions indicators

**Figure 7:**
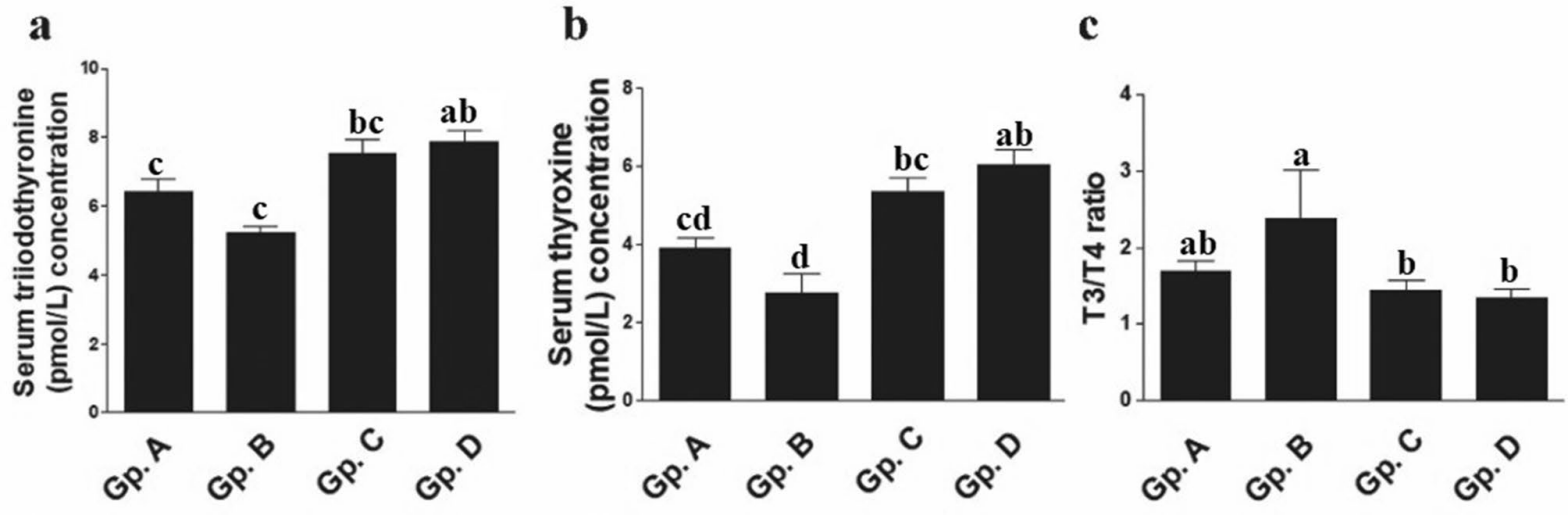
Effect of temperature changes on serum thyroid hormones (**a**: FT3; **b**: FT4; **c**: T3/T4 ratio) in Dabb lizard (*U. aegyptia*). Gp. A: control group; Gp. B: low temperature exposed group; Gp. C: moderate temperature exposed group; Gp. D: high temperature exposed group; FT4: free thyroxine; FT3: free triiodothyronine. ^abcd^Means above each bar with different letters in different Dabb groups i.e. Gp. A, Gp. B, Gp. C and Gp. D, were significantly different (*P* < 0.05).

With except for serum thyroid functions indicators (FT3 and FT4) in high temperature exposed Dabb, the present results revealed no remarkable changes in thyroid functions biomarkers i.e. triiodothyronine (T3), thyroxine (T4) and T3/T4 ratio, between control group, and other treated groups (Low and moderate temperature exposed Dabb lizards). Significant elevations (*P* < 0.05–0.001) in in serum T3 and T4 were demonstrated in high temperature exposed Dabb comparing with control group (Fig. 7).

### Serum oxidant and antioxidant analysis

**Figure 8:**
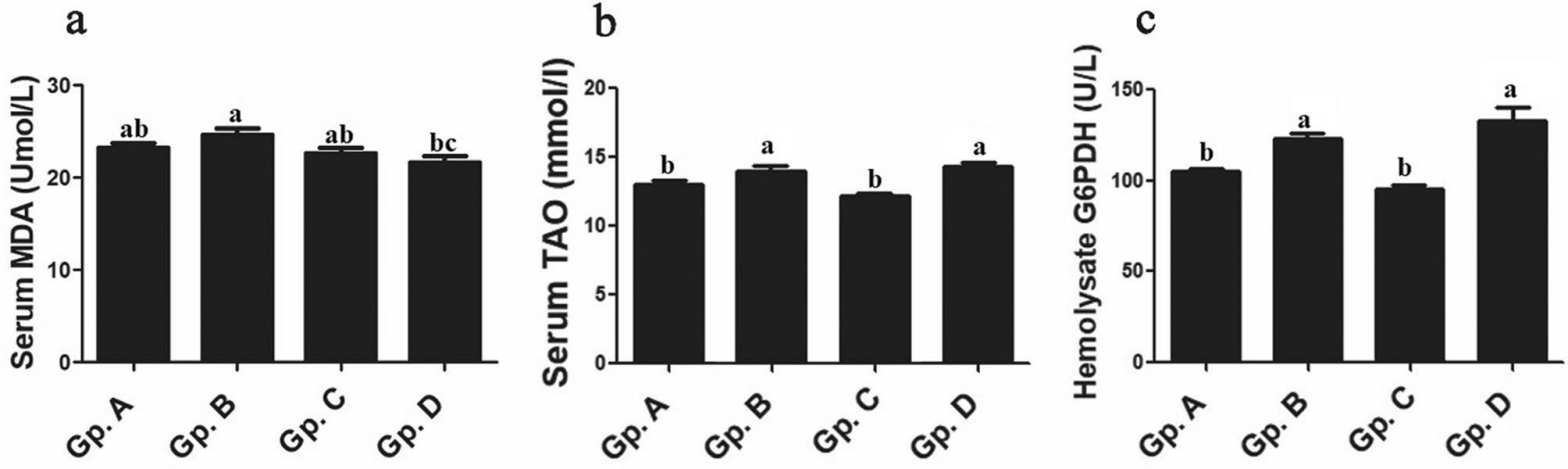
Effect of temperature changes on serum lipid peroxidation, total antioxidant capacity, and G6PDH (as an antioxidant enzyme) activity (**a**: MDA; **b**: TAO; **c**: G6PDH) in Dabb lizard (*U. aegyptia*). Data were expressed as mean ± SE. Gp. A: control group; Gp. B: low temperature exposed group; Gp. C: moderate temperature exposed group; Gp. D: high temperature exposed group; G6PDH: glucose-6-phospate dehydrogenase; TAO: total antioxidant; MDA: malondialdehyde. ^abc^Means above each bar with different letters in different Dabb groups i.e. Gp. A, Gp. B, Gp. C and Gp. D, were significantly different (*P* < 0.05).

The current study mentioned significant changes in serum oxidant/antioxidant parameters whereas serum concentrations of TAO and G6PDH were significantly (*P* < 0.05–0.001) higher in low temperature exposed group and high temperature exposed group than those in control group while these significant changes were absent between moderate temperature exposed one and control Dabb group. On the other hand, different temperature exposed groups had no significant alterations in their serum levels of MDA comparing with control one (Fig. 8).

### Muscle biochemical analysis i.e. muscle metabolic biomarkers

**Figure 9:**
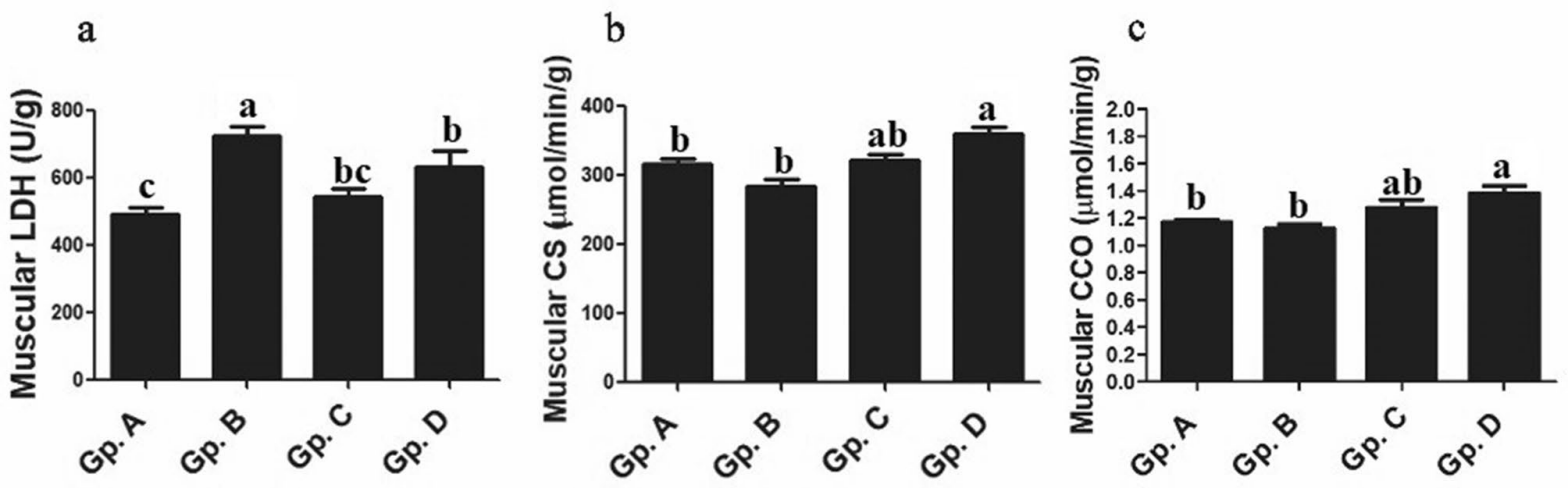
Effect of temperature changes on muscular metabolic enzymes (**a**: LDH; **b**: CS; **c**: CCO) in Dabb lizard (*U. aegyptia*). Data were expressed as mean ± SE. Gp. A: control group; Gp. B: low temperature exposed group; Gp. C: moderate temperature exposed group; Gp. D: high temperature exposed group; CCO: cytochrome c oxidase; CS: citrate synthase; LDH: lactate dehydrogenase. ^abc^Means above each bar with different letters in different Dabb groups i.e. Gp. A, Gp. B, Gp. C and Gp. D, were significantly different (*P* < 0.05).

Regarding to muscle metabolic biomarkers, significant (*P* < 0.05) elevations in muscular LDH, CS and CCO in high temperature exposed Dabb group when they compared with control group. With except for muscular LDH (*P* < 0.001) in low temperature exposed group, these significant changes were not stated between control Dabb lizards group and each of low and moderate temperature exposed groups. Muscular LDA levels were significantly (*P* < 0.001) higher in low temperature treated Dabb comparing with control one (Fig. 9).

### Correlations between blood picture indices in investigated Dabb lizards

Positive correlations were reported between RBCs, Hb, MCHC and WBCs in investigated Dabb lizards whereas their concurrently significant elevations were stated in control group and high temperature exposed ones. In contrast, their associatively significant reductions were reported in low and moderate temperature exposed lizards’ groups. Positive correlations were also described between MCV and MCH in investigated Dabb. MCV and MCH values were increased simultaneously in low and moderate temperature exposed lizards’ groups, while their drops were significantly correlated in control group and high temperature exposed ones. On other side, negative correlations were observed between RBCs, Hb, MCHC and WBCs, and each of MCV and MCH in different Dabb groups whereas a significant raise in their values was associated with a remarkable decline in MCV and MCH values. Moreover, positive correlations were also demonstrated between HCT and PLT values in examined Dabb (Table 2).

**Table 2:**
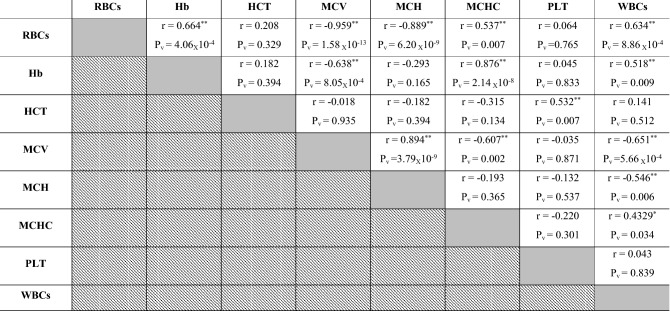
Pearson correlation coefficient between blood picture indices in investigated Dabb lizards.

*RBC* Red blood corpuscles, *Hb* Haemoglobin concentrations, *HCT* Haematocrit value, *MCV* Mean corpuscular volume, *MCH* Mean corpuscular haemoglobin, *MCHC* Mean corpuscular haemoglobin concentration, *PLT* Platelets, *WBC* White blood cell, *R* Correlation.

P_v_: *P* value: *Significant (two-tailed) *p* < 0.05. **Significant (two-tailed) *p* < 0.01. Gray backgrounds referred to correlation between the same parameter e.g. RBCs and RBCs where there was no correlation. Diagonal backgrounds referred that this correlation was previously reported in the previous row e.g. correlation between RBCs and Hb was reported in row 1 and so there was no need to repeat it at row 2.

### Correlations between lipid profiles in investigated Dabb lizards

With except for serum LDL and globulins, positive correlations were reported between most blood lipid profiles in investigated Dabb lizards whereas serum concentrations of glucose, TC, TG, HDL, TPs and albumins were significantly increased simultaneously in control and low temperature exposed Dabb then concomitant reduction in their blood values was observed in moderate and high temperature exposed groups (Table 3).

**Table 3:**
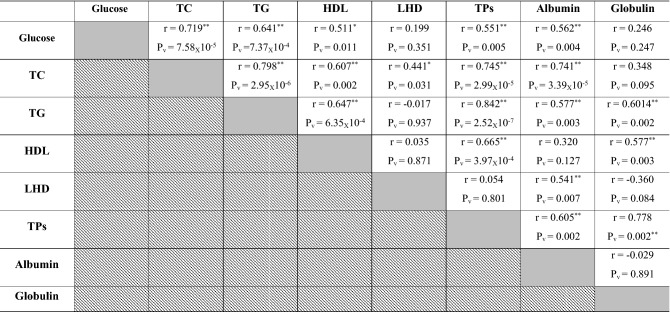
Pearson correlation coefficient between blood lipid profiles in investigated Dabb lizards.

*TC* Total cholesterol, *TG* Triglyceride, *HDL* High-density lipoprotein, *LHD* Low-density lipoprotein, *TPs* Total proteins, *R* Correlation.

P_v_: *P* value: *Significant (two-tailed) *p* < 0.05. **Significant (two-tailed) *p* < 0.01. Gray backgrounds referred to correlation between the same parameter e.g. glucose and glucose where there was no correlation. Diagonal backgrounds referred that this correlation was previously reported in the previous row e.g. correlation between glucose and TC was reported in row 1 and so there was no need to repeat it at row 2.

### Correlations between hepatic biomarkers in investigated Dabb lizards

No significant correlations were reported between serum ALT, AST, ALP and bilirubin as hepatic function indicators in investigated Dabb lizards under different thermal treatments (Table 4).

**Table 4:**
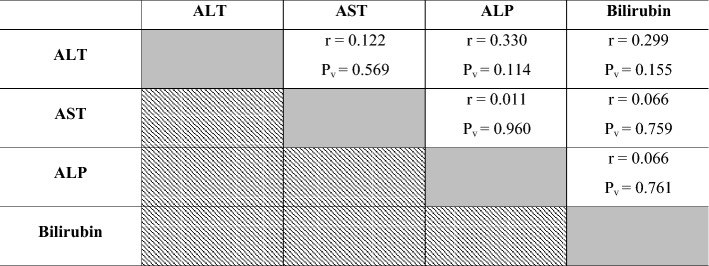
Pearson correlation coefficient between hepatic biomarkers in investigated Dabb lizards.

*ALT* Alanine aminotransferase, *AST* Aspartate aminotransferase, *ALP* Alkaline phosphatase, *R* Correlation.

P_v_: *P* value: *Significant (two-tailed) *p* < 0.05. **Significant (two-tailed) *p* < 0.01. Gray backgrounds referred to correlation between the same parameter e.g. ALT and ALT where there was no correlation. Diagonal backgrounds referred that this correlation was previously reported in the previous row e.g. correlation between ALT and AST was reported in row 1 and so there was no need to repeat it at row 2.

### Correlations between renal indicators, serum electrolytes and thyroid function parameters in investigated Dabb lizards

With except for serum creatinine, the present study stated positive correlations between renal biomarkers, serum electrolytes and thyroid functions parameters whereas serum levels of urea, uric acids, Na^+^, K^+^, Cl^−^, FT3 and FT4 were concomitantly elevated in control, moderate and high temperature exposed Dabb groups. In contrast, their serum values had a simultaneously significant drop in low temperature exposed lizards (Table 5).

**Table 5:**
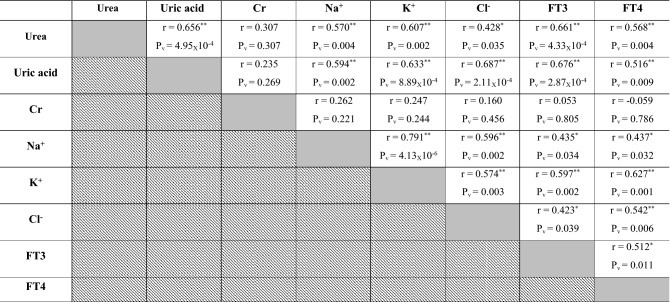
Pearson correlation coefficient between renal indicators, serum electrolytes and thyroid function parameters in investigated Dabb lizards.

*Cr* Creatinine, *Na*^+^ Sodium, *K*^+^ Potassium; *Cl*^*−*^ Chloride, *FT3* Free triiodothyronine, *FT4* Free thyroxine, *R* Correlation.

P_v_: *P* value: *Significant (two-tailed) *p* < 0.05. **Significant (two-tailed) *p* < 0.01. Gray backgrounds referred to correlation between the same parameter e.g. urea and urea where there was no correlation. Diagonal backgrounds referred that this correlation was previously reported in the previous row e.g. correlation between urea and uric acid was reported in row 1 and so there was no need to repeat it at row 2.

### Correlations between serum and muscular oxidant/antioxidant biomarkers in investigated Dabb lizards

The present work revealed no significant correlations between serum oxidant/antioxidant parameters in different Dabb groups with except for positive correlations had been reported between serum TAO and serum G6PDH. Their serum concentrations were concomitantly elevated in low and high temperature exposed groups, while they were simultaneously reduced in the other groups (Table 6).

**Table 6:**
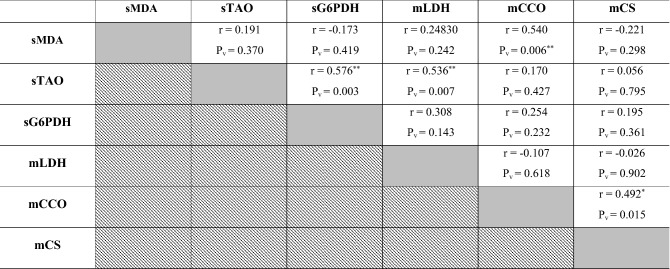
Pearson correlation coefficient between serum and muscular oxidant/antioxidant biomarkers in investigated Dabb lizards.

*sMDA* Serum malondialdehyde, *sTAO* Serum total antioxidant, *sG6PDH* Serum glucose-6-phospate dehydrogenase, *mLDH* Muscular lactate dehydrogenase, *mCCO* Muscular cytochrome c oxidase, *mCS* Muscular citrate synthase, *R* Correlation.

P_v_: *P* value: *Significant (two-tailed) *p* < 0.05. **Significant (two-tailed) *p* < 0.01. Gray backgrounds referred to correlation between the same parameter e.g. sMDA and sMDA where there was no correlation. Diagonal backgrounds referred that this correlation was previously reported in the previous row e.g. correlation between sMDA and sTAO was reported in row 1 and so there was no need to repeat it at row 2.

The present work also revealed no significant correlations between muscular oxidant/antioxidant parameters in different Dabb groups with except for positive correlations had been stated between muscular CCO and muscular CS. Their values were concurrently increased mainly in high temperature exposed Dabb while they were concomitantly dropped mainly in control one (Table 6).

No significant correlations were demonstrated between serum and muscular oxidants/antioxidants parameters except for positive correlation between serum MDA and muscular CCO, and for that between serum TAO and muscular LDH (Table 6).

## Discussion

### Clinical findings and morphometric measurements

Monitoring reptile thermoregulation is becoming increasingly important for determining the impact of climate change on various species of reptiles as well as predicting whether or not they will adapt to local warming trends. The current results add that clinical findings and morphometric measurements including body weight, TBL (SVL plus TL) and BMI have no significant alterations in all iterated Dabb lizards’ groups. Sinervo et al.^30^ observe a decrease in lizard biodiversity because of local warming trends and changed thermal niches. Reptile blood has a wealth of information on how they have adapted to metabolic variations during environmental changes.

### Complete blood picture indices

Alterations in haematological indices usually reflect environmental changes and diseased conditions, and so they are of high efficacy in assessing the systemic changes^24^. The ability of RBCs to counteract oxidative stress was greatly dependent on G6PDH^25^. Referring to the present work, the estimated blood pictures parameters in Gp. C are not remarkably changed comparing to control group. All investigated Dabb lizards’ groups have no significant changes for PLT and mean corpuscular values between control group and treated Dabb lizards’ groups. In contrast, RBCs, Hb and WBC are remarkably raised in low and high temperature exposed lizards in comparison with control one. Moreover, these significant changes are reported for HCT only between high temperature exposed group and control group while they are absent for low temperature exposed group. These results are supported by Said and Hussein^24^ that report a significant drop in some haematological parameters including RBCs, HCT and Hb as well as serum glucose level in *U. aegyptius* during winter weather. Furthermore, Maclean et al.^31^ show that RBCs, HCT, and Hb are related to the habit of the animals as they are low in comparatively sluggish forms. At low temperatures, the metabolic rate and energy turnover in reptiles are reduced since all physiological processes have declined. With referring to the current study, the increase in some haematological of Dabb lizard exposed to low and high temperature are in agreement with other studies^32–36^ that explain the findings as a result of anoxic environments (hypoxia) or/and dehydration. At the end of the hibernating period, Souza et al.^37^ discover a rise in the count of reticulocytes (immature RBCs) is attributable to erythrocyte regeneration. So, the present work considers this increase as one of the lizards' adaptations to the harsh environment to survive. Al-Johany and Haffor^38^ observe that WBC and lymphocyte counts are significantly increased in lizards exposed to 33–35 °C compared with the control animals.

### Serum Lipid profile indices

Several studies report a significant decline in serum levels of glucose, cholesterol, and urea in serum samples at low temperature (2 °C) or during winter periods (8–15 °C)^5,24,31,39^. During winter brumation, reptiles use carbohydrates as the main source of energy and a decline in general metabolism that usually reflects a reduction in glycolytic capacity^40^. A shift from carbohydrate to lipid metabolism can offset the lower rates of flux through glycolysis under aerobic conditions^40^. Marked hypoglycemia in reptiles may refer to onset of fat and glycogen store in its tissues, at low temperatures. During the winter months, reptiles can maintain activity through a combination of behavioural thermoregulation and/or compensation of metabolic capacity at the molecular and cellular levels^6–8^. The current study mentions no significant changes for serum Lipid indices including HDL, LDL, atherogenic index (TC/HDL and LDL/HDL), globulin and A/G ratio between different treated Dabb lizards’ groups and control one. Different treated groups of the investigated Dabb lizards show remarkably changes for blood glucose, TC, TGs, TPs and albumins comparing to control Dabb group. Significant reductions of serum glucose, TC, TGs, TPs and albumins are reported in low temperature exposed group. In contrast, moderate and high temperature exposed groups have significantly higher levels of blood glucose, TC, TGs, TPs and albumins. These results are in consistent with the previous reports which have revealed a remarkable drop in some blood lipid and electrolytes parameters such as TC, TGs, albumin, glucose, and Na^+^ of Dabb lizard (*U. aegyptia*) exposed to low temperature after one week^5,24,39^ at the same time as muscle LDH activities elevated. The other reports have added that hypoglycaemia was reported in reptiles during winter, which may be due to the reduction in the nervous system activity during brumation^41^, that can lead to increase the levels of the inhibitory neurotransmitter such as gamma-aminobutyric acid (GABA) in the brain of animals may contribute to metabolic depression^42^. It has been suggested that the cryoprotective action of glucose protected membranes and enzymes against damage related to low temperatures. In *Uromastyx aegyptius* species, prevention of freezing of intracellular fluid is critical because the intracellular ice crystals can destroy the integrity of intracellular plasma membranes of organelles^43^. Cholesterol may also have a cryoprotective effect and the alterations in the levels of cholesterol in the blood have been noted in this work support the idea that cholesterol has been involved in physiological thermoregulation. It seems that the lipid content of tissues requires adjustment to ensure that regular function is preserved at reduced body temperature.

### Serum hepatorenal biomarkers and electrolytes indices

The current work does not report significant variations for hepatic functions indicators whereas serum activities and values of bilirubin, ALT, AST and ALP are not remarkably altered between control group and other thermal treated Dabb lizards’ groups (Moderate and high temperature exposed groups). On the other hand, the previous observations on reptiles add that values of ALT, bilirubin and creatinine levels are of low quantities in Dabb lizard tissues. These changes are usually considered to be of little clinical significance in reptiles. Bilirubin and creatinine may not be produced at all in most reptiles^44,45^.

The renal biomarkers show significant alterations for serum urea and uric acid between control group and some thermal treated lizards groups (Moderate and high temperature exposed groups) meanwhile these changes are absent between control group and low temperature exposed group. Serum concentrations of urea and uric acid are significantly elevated in moderate and high temperature exposed Dabb comparing with their control values. On other hand, serum concentrations of Cr have no significant variations between control group and other treated Dabb groups. These results are confirmed by the previous studies in which the animals have been exposed to increased ambient temperatures, have displayed higher metabolic rate so that blood urea and glucose (As a source of energy for intracellular metabolism), are found to be significantly increased^5,8^. The increased levels of blood glucose, TPs, urea, and uric acid in lizards with exposure to elevated temperatures are usually attributable to plasma volumes reduction^46,47^. In addition, the increase in urea and TPs levels may be attributed to a rise in urea-generating protein metabolism, an increase in nitrogen retention or excessive protein breakdown from the muscles to the blood^5,8^. An important function of serum protein is the maintenance of the normal distribution of body water by controlling the osmotic balance between the circulating blood and the membrane of tissues, as well as the transport of lipids, hormones, and inorganic materials^45^. Referring to the present study, it reports an increase in some biochemical and electrolytes parameters such as TC, TGs, TPs, glucose, urea, uric acids, TAO, G6PDH, Na^+^ and K^+^ levels of Dabb lizard exposed to high temperature that in turn are in consistent with these previous reports^24,32,39,48^. After feeding in active forms, TPs rise higher^39^. The primary products of protein catabolism in reptiles are uric acid and urea, this may provide an explanation for rise in their concentration.

Serum electrolytes values of Na^+^ and K^+^ in the present work have significant changes between control Dabb lizards group and treated groups (Low and high temperature exposed groups), however, these significant variations are not observed between control group and moderate temperature exposed Dabb lizards. Comparing with control group, significant elevations in blood concentrations of Na^+^ and K^+^ in high temperature exposed Dabb group are demonstrated while these parameters are significantly dropped in low temperature exposed group. Moreover, no remarkable changes are stated between control Dabb lizards and the other treated groups for serum values of chloride. These results are confirmed by the previous reports which reveal a remarkable drop in some blood electrolytes parameters such as Na^+^ of Dabb lizard (*U. aegyptia*) exposed to low temperature after one week^5,39^. At low temperatures, ion transport is inhibited, resulting in a decrease in plasma Na^+^. Short-term cold exposure causes clear drop in blood Na^+^ and K^+^ values in the snake *Natrix* and the lizard *Anolis*^49,50^. Na^+^ is usually the main contributing factor for osmotic pressure in the internal environment. The movement of Na^+^ into intracellular fluids, as well as likely Na^+^ losses through the kidney, whose tubules decreases their ability to resorb Na^+^ when chills, contributes to such alterations^32^. The release of Na^+^ from intracellular spaces acts as a buffer for lactate formation. Lactate levels in the blood are high due to anaerobic glycolysis. But it returns to resting levels most quickly in lizards at their optimal body temperature^32^. According to Moberly’s^51,52^ reports on the lizard i.e. *Iguana iguana*, anaerobic metabolism (The breakdown of glycogen or glucose into lactic acid) may be particularly significant in reptiles. Compared to aerobic pathways, the anaerobic mechanism produces comparatively little energy^53^. Since anaerobic energy production in reptiles is essentially temperature independent, it allows for activity at all body temperatures and does not demand a high resting metabolism^53^. In the lizards *Trachydosaurus rugosus* and *Agama agama*, as well as the snake *Natrix natrix*, the adrenal gland regulates Na^+^ and K^+^ levels^54^. Reptiles can withstand a wide range of hydration levels. Hypernatremia (rise in serum Na^+^ level) and hyperkalaemia (Rise in serum K^+^ level) report in many species of lizards during the hot dry season may be as result of dehydration^55–57^. Water loss through the kidneys is negligible in the summer, but salt levels rise due to evaporation^32^.

### Serum thyroid functions indicators

With except for serum thyroid functions indicators (FT3 and FT4) in high temperature treated Dabb, the present results reveal no remarkable changes in thyroid functions biomarkers i.e. T3, T4 and T3/T4 ratio, between control group and other treated groups (Low and moderate temperature exposed groups). Significant elevations in serum T3 and T4 are demonstrated in high temperature exposed group comparing with control one. These results have been confirmed by Sciarrillo et al.^20^; John-Alder^58^. Furthermore, changes in thyroid hormone levels in the blood stream may also play a role in seasonal metabolic upregulation of enzyme activity. Sinha and Choubey^19^ also report a maximum level of activity was displayed by the thyroid gland of *U. harrdwickii*, the Indian spiny-tailed lizard at elevated ambient temperature. Furthermore, other researches on lizards have demonstrated the existence of a correlation between T4 elevation and high temperature^20^ as well as high plasma aerobic enzyme activity (LDH, CCO, and CS) and a general increase in aerobic capacity and metabolic rate^58,59^.

### Oxidant and antioxidant analysis

An increase in ROS production and antioxidants has already stated during high temperature (42 °C) exposure^15^. An important normal physiological defence mechanism is the generation of ROS by WBCs. High levels of oxygen free radicals can be successfully controlled through antioxidant enzyme activation^17^. Indeed, Afifi and Alkaladi^60^ confirm that free radicals were produced when the use of oxygen by mitochondria gets raise by 0.1%. The current study mentions significant changes in serum oxidant/antioxidant parameters whereas serum concentrations of TAO and G6PDH are significantly higher in each of low temperature and high temperature exposed lizards’ groups than those in control one while these significant changes are absent between moderate temperature exposed ones and control group. On the other hand, different thermal treated groups of investigated Dabb in the present study have no significant alterations in their serum levels of MDA comparing with control group. In the study, low temperature has no effect on oxidant/antioxidant parameters. Meanwhile, previous studies have indicated a significant decrease in serum free radicals and increase in non-enzymatic antioxidant (vitamin C) during brumation^38,60^. In the present study, MDA level (as marker for lipid peroxidation) is not affected and concomitant with an increase in serum G6PDH, TAO capacity, and WBC. G6PDH as an antioxidant enzyme catalyse the conversion of G6P to 6-phosphogluconate with the generation of NADPH. G6PDH usually which provides NADPH for lipogenesis and cholesterogenesis, and ribose for the synthesis of nucleic acids and thus it is considered a key enzyme in the pentose phosphate pathway. NADPH serves as an important cofactor for the preservation of GSH, which has a role in ROS scavenging^47^. Cellular ROS accumulates at a lower rate when G6PD activity is increased, so that GSH stores are maintained, and oxidative stress is suppressed^60^. A study on *U. aegyptia* finds that moderate heat stress of 33–35 °C has intensified antioxidant enzyme activity and caused immune cells to proliferate and differentiate^38^. Hence, it can be said that *U. aegyptia* has protection not only against the damaging effects of free radicals owing to its robust total antioxidant defence system but also against infections owing to its strong immune system, particularly when exposed to elevated temperatures.

### Muscle biochemical analysis i.e. muscle metabolic biomarkers

Regarding to muscle metabolic biomarkers, the current work states significant elevations in muscular LDH, CS and CCO in high temperature exposed group comparable with control Dabb lizards group. With except for muscular LDH in low temperature exposed group, these significant changes are not stated between control lizards, and each of low and moderate temperature exposed groups. Muscular LDA levels are significantly higher in low temperature exposed Dabb comparing with control one. Our results have indicated that muscular LDH activity are significantly higher at low temperature than at high temperature, possibly due to the high need to compensate for the metabolic depression effect of lower body temperatures. These data are in agreement with those of earlier studies^48,61^, which have suggested that increased muscle enzymes levels may have important implications for reptilian thermal physiology. LDH usually catalyses the reversible oxidation of lactate to pyruvate with the cofactor Nicotinamide adenine dinucleotide (NAD; anaerobic glycolysis). It may play a key role in energy production of anaerobic metabolism to prolong lizard survival especially at low temperature. Furthermore, it is essential to generate NAD+ so that glycolysis could be sustained. At low temperatures, several reptile species display heightened thermal sensitivity of metabolic and physiological processes, which contribute to a decrease in activity levels and whole animal metabolism^62^. The biochemical acclimatization is an important in physiological thermoregulation of reptiles. The current results have revealed a significant increase in the levels of muscular metabolic enzymes like LDH, CS, and CCO in the groups with high temperature exposure^7,19^. LDH is an important enzyme in anaerobic glycolysis, while CS is an essential enzyme in Krebs cycle (or for CAC or tricarboxylic acid cycle; TCA), whereas CCO (complex IV; transmembrane protein complex) is the primary regulatory enzyme in the electron transport chain (or for oxidative phosphorylation). There is evidence that the ambient temperature may influence the CS level in ectotherms^2,44^. The condensation between the acetyl methyl group of acetyl CoA and the carbonyl of oxaloacetate has been catalysed by CS to produce citrate as part of CAC. Elevation of CS levels explains the seasonal changes, which is known to occur during seasonal acclimatisation of ectotherms^7^. CCO is considered an important marker for the prevention of excessive build-up of ROS. The previous studies on the yellow-belly gecko (*Hemidactylus faviviridis*), hardwick's spiny-tailed lizard (*U. hardwickii*)^63^ and spiny tailed lizard (*U. aegyptia*)^5^ report that the levels of metabolic enzymes in the blood increased during the activity period compared with the period of brumation of those species of reptiles. Furthermore, other researches on lizards have demonstrated the association between high temperature as well as high plasma aerobic enzyme activity (LDH, CCO, and CS) and a general increase in aerobic capacity and metabolic rate^20,58,59^.

### Correlations between different laboratory indices in investigated Dabb lizards

Positive correlations have been reported between RBCs, Hb, MCHC and WBCs in investigated Dabb lizards. Positive correlations are also described between MCV and MCH in investigated Dabb. On other side, negative correlations are observed between RBCs, Hb, MCHC and WBCs, and each of MCV and MCH in different Dabb groups. With except for serum LDL and globulins, positive correlations are reported between most blood lipid profiles including glucose, TC, TG, HDL, TPs and albumins, in investigated Dabb lizards. The present study mentions no significant correlations between serum ALT, AST, ALP and bilirubin as hepatic function indicators in investigated Dabb lizards under different thermal treatments. With except for serum creatinine, the present study states positive correlations between renal biomarkers (Urea and uric acids), serum electrolytes (Na^+^, K^+^ and Cl^−^) and thyroid functions parameters (FT3 and FT4) as they are concomitantly elevated in control, moderate and high temperature exposed Dabb. In contrast, their serum values have a simultaneously significant drop in low temperature exposed lizards. The previous articles have clarified one of the possible explanations for this remarkable increase in blood composition indicates the adaptation of lizards to environmental stressors to maintain the highest levels of oxygen in their bodies and also to maintain the integrity of blood cells. Also, the noticeable increase in the levels of sugar, fats, protein, as well as thyroid hormones, kidney functions, and metabolic enzymes indicates an increase in metabolic rates in proportion to the difference in temperature to keep the lizard alive and healthy. According to Snyder and Weathers^64^, the volume of oxygen consumed by reptiles is typically proportional to the ambient oxygen pressure, and metabolism rises with increasing temperature^65^. On the other hand, reptile fluid and electrolyte balance is greatly influenced by body temperature, which also affects the physical characteristics of electrolytes and the metabolic rate of the organisms. During eating, temperature fluctuations, and osmotic stress, the potassium and sodium levels in the reptiles vary, significantly changing the electrolyte balance^32,66^. In addition, the lack of relationships between liver enzymes and bilirubin indicates the safety of liver cells and their normal functions. In contrast, biochemical processes in ectotherms slow down with ambient low temperatures, which results in less oxidant generation^64,67,68^. In addition, many species hibernate in cold climates, which is a period when their metabolism as well as partial oxygen pressure are reduced to a minimum^68^. We can explain that the differences in haematological, lipids/protein profiles between animals from low and high exposure of temperature are due to higher metabolic rate, thyroid hormones, and enzymatic antioxidant defences in high temperature group than in low temperature group. The present work reveals no significant correlations between serum oxidant/antioxidant parameters in different Dabb groups with except for positive correlations have been reported between serum TAO and serum G6PDH. No significant correlations are described between muscular oxidant/antioxidant parameters in different Dabb groups with except for positive correlations have been stated between muscular CCO and muscular CS. Finally, no significant correlations are demonstrated between serum and muscular oxidants/antioxidants parameters except for positive correlation between serum MDA and muscular CCO, and for that between serum TAO and muscular LDH. TAO is an indicator of endogenous antioxidant defence system against lipid peroxide formation and oxidative stress. The possible explanation for this result is that lizards have a strong antioxidant system^17^ and immune system^69^ that makes them have the ability to withstand the difficult thermal environment that they are exposed to in the laboratory and thus confront any oxidative stress biomarker with the increase of electron transfer enzymes during metabolism and enzymes that indicate cell toxicity. In fact, it has already been demonstrated that free radical production in ectotherms rises with higher metabolism and temperature^67,70^.

## Conclusions

The Dabb lizard (*U. aegyptia*) has been exposed to adaptations in physiological and biochemical aspects to maintain homeostasis in response to environmental temperature changes as a protective response against free radicals (especially at high temperature exposure) to maintain normal function and to survive in arid/harsh environments against heat stress. There are possible mechanisms for physiological thermoregulatory of this ectotherm that include increased concentrations of metabolic enzymes in muscle, changes in thyroid hormones levels and improvement of their antioxidant defense system. Dabb lizards are capable to upregulate antioxidant defense in response to increasing temperature, protecting the organism from oxidative stress. However, we do not know if there are long-term costs for thermal acclimation. The upregulation of endogenous antioxidants may come at a fitness cost because it requires ATP consumption, with possible negative implications for reproduction/survival. Moreover, *U. aegyptia* does not undergo oxidative stress because it has an effective antioxidant defense mechanism that guards it from the damaging effects of free radicals either during low or high temperature exposure. Different significant correlations have been stated between variably estimated laboratory indices in the investigated Dabb lizards under different thermal treatments. The specific mechanisms have been used by these lizards to survive the consequences of climate change are yet unclear. Therefore, additional research must be conducted to understand how physiologically/biochemically thermoregulation can be controlled in reptiles in *U. aegyptia*.

## Data Availability

The datasets used and/or analysed during the current study available from the corresponding author on reasonable request.

## Abbreviations

A/G ratio: Albumin/globulin ratio
ALP: Alkaline phosphatase
ALT: Alanine transaminase
ATP: Adenosine triphosphate
AST: Aspartate aminotransferase
BMI: Body-mass-index
CAC: Citric acid cycle
CCO: Cytochrome c oxidase
CS: Citrate synthase enzyme
FT3: Free triiodothyronine
FT4: Free thyroxine
G6PDH: Glucose-6-phosphate dehydrogenase
GSH: Reduced glutathione
Hb: Haemoglobin
HCT: Haematocrit value
HDL: High-density lipoproteins
LDH: Lactate dehydrogenase
LDL: Low-density lipoproteins
MCH: Mean corpuscular haemoglobin
MCHC: Mean corpuscular haemoglobin concentration
MCV: Mean corpuscular volume
MDA: Malondialdehyde
Na^+^: Sodium
NAD: Nicotinamide adenine dinucleotide
NADPH: Nicotinamide adenine dinucleotide phosphate
PLT: Platelets
K^+^: Potassium
RBCs: Red blood corpuscles
ROS: Reactive oxygen species
TAO: Total antioxidant status
TBL: Total body length
TC: Total cholesterol
TCA: Tricarboxylic acid cycle
TGs: Triglycerides
T4: Thyroxine
T3: Triiodothyronine
TPs: Total proteins
WBC: White blood cells
